# Comparison of Chest Computed Tomography (CT) Imaging Patterns and Severity among COVID-19 Patients during the First and Fourth Waves in Addis Ababa, Ethiopia

**DOI:** 10.1155/2023/6385162

**Published:** 2023-11-29

**Authors:** Lensa Million Baharu, Amir Alwan, Seife Teferi Dellie, Tesfaye Kebede Legesse, Kibruyisfaw Weldeab Abore

**Affiliations:** ^1^Department of Radiology, College of Medicine and Health Sciences, Addis Ababa University, Addis Ababa, Ethiopia; ^2^Department of Pediatrics, Yirgalem Hospital Medical College, Yirgalem, Sidama, Ethiopia

## Abstract

**Background:**

Studies done globally had shown that chest imaging patterns of Corona virus disease 2019 (COVID-19) infection varied depending on the strains of the virus and the waves of the pandemic. There is no published literature done in Ethiopia to examine whether there is any difference in chest computed tomography (CT) findings of COVID-19 patients during the first and fourth waves. Thus, this study tries to fill the gap of knowledge in that regard.

**Objective:**

To describe and compare chest CT scan imaging pattern and assess the predictors of chest CT severity of the first and fourth wave of COVID-19 infection.

**Methods:**

An institution-based cross-sectional study was conducted on 200 polymerase chain reaction test confirmed COVID-19 patients who underwent chest CT scan imaging in two diagnostic centers in Addis Ababa city. Pioneer and Wudassie diagnostic centers were selected due to the high case load and availability of well-experienced cardiothoracic radiologists. Data were collected from July 1 to August 3, 2022, using a structured Google form sheet questionnaire. Binary logistic regression was performed, and statistical significance was assessed at a level of significance *α* = 0.05.

**Results:**

Comparatively higher proportion of patients from the first wave had positive chest CT finding than fourth wave (99% vs. 69%). Bilateral lung involvement and lower lobe predilection were seen for both waves of COVID-19. Ground glass opacity and consolidation were the most common CT features for both waves. Delayed chest CT features such as traction bronchiectasis were primarily seen among first-wave patients. Mean global CT severity score was higher for the first-wave patients (13.18 vs. 8.31), and the mean difference is statistically significant (*p* < 0.001). Duration of symptoms was a statistically significant predictor of CT severity during the first wave of COVID-19, and patients that presented later than 14 days had 4.12 times higher odds of being in the severe CT score category than those that presented less than 7 days (AOR = 4.12, *p* = 0.011). There was no statistically significant predictor of CT severity for the fourth wave in this study.

**Conclusion:**

Chest CT positivity was comparatively higher for first wave patients. Common features included bilateral involvement, lower lobe involvement, ground glass opacity, and consolidation. Mean chest CT severity was comparatively higher for the first wave than the fourth wave, and the duration of symptoms was a statistically significant predictor of the CT severity for first wave.

## 1. Introduction

Novel coronavirus disease 2019 (COVID-19) is a viral infectious disease that was first detected in Wuhan, Hubei Province of China in 2019 [1]. It is a multisystem syndrome with a spectrum of presentation ranging from asymptomatic disease to life-threatening complications requiring invasive management like extracorporeal membrane oxygenation [2]. More than 489 million confirmed cases with 6 million deaths were reported globally as of December 2022 [3].

The first case in Ethiopia was identified on March 13, 2020 [4]. There were four waves of COVID-19 based on the epidemiologic curve. The first wave of COVID-19 lasted from June to November 2020, while the fourth wave lasted from December to February 2022. Although genomic sequencing was not available, the first wave was attributed to the alpha variant, and the fourth wave was attributed to the omicron variant [5].

Reverse transcriptase polymerase chain reaction (RT-PCR) performed on nasopharyngeal and oropharyngeal swab is the gold standard for diagnosing COVID-19 [6]. However, chest CT has become an important diagnostic tool for COVID-19 when used in close combination with clinical presentation and epidemiological evidence for the disease. Furthermore, chest CT has also been shown to be useful for the management of COVID-19 patients as well as differentiating various diseases that COVID-19 could mimic [2]. A study done by Fang et al. observed that the sensitivity of CT in diagnosing COVID-19 was very higher than that of RT-PCR [7].

Over the past years, chest imaging findings of the COVID-19 infection have been described in details. On chest imaging, the lungs could appear normal. However, studies conducted globally had shown that some of the commonest radiological findings included ground glass opacities on plain radiographs and ground glass attenuation on CT scans, focal or diffuse areas of consolidation, and septal thickening most commonly noted in the lower lobes. Bilateral lung changes were also more common than unilateral lung involvement. Atypical findings of COVID-19 infections described in literature include pleural and pericardial effusions, calcifications, pneumothorax, cavitation, lung collapse, mediastinal enlargement, and cardiomegaly [8–11]. Based on previous studies, chest CT features were shown to vary based on age of the patients, duration of symptom at presentation, COVID-19 vaccination status, and the strain of the virus [12–15]. A study done in Ghana had demonstrated that more patients in the second wave had evidence of COVID-19 disease on chest imaging than those patients in the first wave [8]. Furthermore, a study done Italy in 2021 had shown that, although there was no difference in the typical chest finding among the first four waves of COVID-19, there was a significant difference in the occurrence of atypical CT features including pleural effusion [16]. Recognizing the differences in chest CT imaging between various waves COVID-19 is vital to diagnosing and treating patients.

This study, to the best of the investigators' knowledge, is the first study done in Ethiopia with the objective of describing and comparing chest CT imaging finding as well as assesses the predictors of chest CT severity among confirmed COVID-19 patients during the first and fourth wave. Therefore, the study would provide useful information and fill the gap of knowledge on CT features.

## 2. Methods and Materials

### 2.1. Study Setting and Study Design

The study was conducted in Addis Ababa city, the capital city of Ethiopia, using a data collected from Pioneer and Wudassie diagnostic centers. Among health facilities with a capacity to perform CT scan imaging, Wudassie diagnostic center and Pioneer diagnostic center were purposively selected due to the high caseload, quality of CT scans (128 slices), and the availability of highly experienced radiologists. Furthermore, in both institutions, chest CT reading was conducted by experienced cardiothoracic radiology subspecialists. Both centers provided imaging and other diagnostics services to dedicated COVID-19 treatment centers and other health facilities.

Noncontrast chest CT scan in standard dose was acquired in volumetric mode, scanning extending from thoracic inlet to caudally include upper abdomen. The acquired CT images are reconstructed into soft tissue lung and mediastinal window and in 1.2–1.5 mm section thickness for interpretations. Contrast enhanced CT angiography was done for those suspected of having pulmonary thromboembolism. A comparative cross-sectional retrospective study design was conducted from July 1, 2022 to August 3, 2022.

### 2.2. Study Population

All patients with confirmed COVID-19 who underwent chest CT scan imaging in the selected health institutions during the first and fourth waves in Addis Ababa city.

### 2.3. Inclusion and Exclusion Criteria

#### 2.3.1. Inclusion Criteria

All patients with PCR confirmed COVID-19 infection that underwent chest CT scan evaluation during the first and fourth waves of COVID-19.

#### 2.3.2. Exclusion Criteria

Patients whose chest CT imaging was not reviewed due to poor quality image to interpret and those with incomplete data were excluded from this study.

### 2.4. Sampling Technique and Sample Size

All patients who underwent a chest CT scan with a confirmed COVID-19 pneumonia were retrieved from the archives of the two institutions, and 240 patients with confirmed COVID-19 cases were found. Among them, 130 patients were from the first wave, and 110 patients were from the fourth wave. After applying the exclusion criteria, however, a total of 210 were eligible candidates (108 from the first wave and 102 from the fourth wave). Subsequently, a 1 :  1 proportion of 100 patients from each wave was selected for comparability.

### 2.5. Data Collection Instruments and Techniques

A structured questionnaire was developed after reviewing various pieces of literature. The questionnaire was then converted in to a Google form sheet. The selected patient's electronic records and chest CT images were retrieved from both diagnostic centers. The data from patient's records and chest CT images were filled into the preprepared Google form using a mobile phone.

### 2.6. Data Processing and Analysis

The collected data were assessed for completeness and exported to the statistical package for social science (SPSS) v.20 and after data cleaning, analysis was done.

Categorical variables were summarized using frequency and percentage, while continuous variables were summarized using mean and standard deviation after testing for normality of data using the Kolmogorov–Smirnov test. Subgroup analysis was performed to assess difference in CT imaging character among various groups.

Association between variables was assessed using chi-square (*χ*^2^) and Independent sample *t*-test was performed to assess differences in mean CT score. Simple binary logistic regression was performed to assess predictors of chest CT severity and those variables with a *p* value of <0.25 were considered as candidate for multivariable logistic regression to determine predictors of chest CT severity, using *α* = 0.05 as the significance level. The association was measured using odds ratio with the corresponding 95% confidence interval.

### 2.7. Operational Definitions

Confirmed COVID-19 patients were defined as those patients with a laboratory (RDT or RT-PCR) confirmed COVID-19 infection [17].

The chest CT severity score was assessed using a semiquantitative CT severity scoring adopted from Gurumurthy et al. [18]. It was calculated based on the involvement of the 5 lobes considering the anatomic extent and the involvement of each lobe. It is scored as follows: 0, no involvement; 1 = <5% involvement; 2 = 5–25% involvements; 3 = 26–50% involvements; 4 = 51–75% involvement; and 5 = >75% involvements. The global CT score was the sum of each lobar score graded from 0 to 25. A score of <7 is classified to be mild, between 8 and 17 moderate, and >18 as severe. For analysis purposes, the score was dichotomized in to severe and nonsevere chest CT score. Those with scores <18 were classified as nonsevere and those with score ≥18 were classified as severe CT score.

Typical chest CT features are common chest CT findings among COVID-19 patients including ground glass opacity, consolidation, broncho-vascular thickening, crazy paving, traction bronchiectasis, and halo sign [8–11].

Atypical chest CT features are uncommon chest CT features among COVID-19 patients including pleural and pericardial effusions, calcifications, pneumothorax, cavitation, lung collapse, mediastinal enlargement, and cardiomegaly were classified as atypical [8–11].

### 2.8. Ethical Statement

Ethical approval was obtained from research and ethics committee of the department of radiology. Permission was requested and granted from the respective institutions to access the medical records of the patients and to retrieve review the patients imaging study for the research purpose only. A written informed consent was waived due to secondary nature of the data. However, no identifiers were included in the data and patients were not contacted for further follow-up. In addition, all the data were kept confidential and only available to the investigator. The data would be discarded appropriately after the objective of the study is achieved.

## 3. Results

### 3.1. Sociodemographic Characteristics of the Participants

Among the 200 COVID-19 confirmed patients that participated in this study, majority of them were greater than fifty years of age (43% vs. 62% for first wave and fourth wave, respectively). Furthermore, more than half of the study subjects were male patients for both groups (53% vs. 69% for the first wave and fourth wave, respectively).

### 3.2. Comorbidity Status

Based on comorbidity status, less than half of the study participants (44%) that had undergone CT imaging during the first wave had comorbid illness, while around two-third (63%) of those participants from the fourth wave had associated comorbidity. Moreover, higher proportions of patients with two or more comorbidity were seen during the fourth wave compared to the first wave (8% vs. 36% during the first and fourth wave, respectively). Hypertension (23% vs. 30%) and diabetes mellitus (22% vs. 29%) were the most common comorbidities in both waves of COVID-19. However, there were comparatively higher proportion patients with comorbid underlying lung disease (2% vs. 11%), renal disease (1% vs. 7%), cardiac illness (0% vs. 16%), and malignancy (0% vs. 9%) during the fourth wave than the first wave (Table 1).

### 3.3. Clinical Symptom of the Participants

More than two-third (74%) of the participants during the fourth wave of COVID-19 presented within the first one week of symptom onset, while around half (59%) of patients from the first wave presented in the first one week of symptom onset. Patient presentation later than 14 days was more common among patients from the first wave compared to fourth wave (27% vs 16%, respectively). However, the difference was not statistically significant (*p* = 0.075).

Cough and shortness of breath were the most common presenting symptoms, and there was no statistically significant difference between the two waves. The study had also shown that there were significantly lower proportions of patients that presented with symptoms of arthralgia/myalgia (3% vs. 33%) and sore throat (2% vs. 49%) during the first wave compared to the fourth wave (*p* < 0.001) (Table 2).

### 3.4. Chest CT Protocols and Findings of COVID-19 Patients

The majority of patients had undergone chest CT angiography on both waves with a slightly higher proportion during the first wave (61% vs. 50% for first wave and fourth wave, respectively). A comparatively higher proportion of COVID-19 patients had positive chest CT finding in the first wave than the fourth wave with 99% CT positivity in the first wave compared to a 69% in the fourth wave of COVID-19 and it was statistically significant (*p* value <0.001) (Table 3).

**Figure 1:**
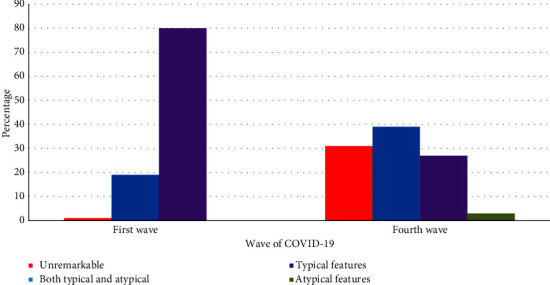
Chest computed tomography (CT) features comparison between the first and fourth waves of COVID-19.

Among patients that had positive chest CT features, a significant majority (80%) had typical features alone, followed by mixed features (both typical and atypical features), while there were no patients with atypical features alone. However, mixed features followed by typical features were the most common CT feature among patients with positive findings during the fourth wave (Figure 1).

Among patients that presented with positive CT finding, a higher proportion of patients (87.9%) during the first wave presented with two or more chest CT findings simultaneously compared to those during the fourth wave (78.3%), but it was not statistically significant (*p* = 0.095). Regarding the symmetry of lung involvement, bilateral CT finding was the most prevalent feature on patients from both COVID-19 waves. Furthermore, a higher proportion of bilateral lung involvement was seen among patient in the first wave (*p* = 0.02).

In relation to the zones of distribution, there was a statistically significant difference in distribution (*p* = 0.004) with peripheral distribution as the most common presentation during the first wave (58; 58.6% vs. 25; 36.2%), while diffuse lung involvement was the most common for the fourth wave (39; 39.4% vs. 37; 53.6%). Central distribution was the least common feature for both waves.

While a comparable proportion of lung lobe involvement was seen in the first wave (right upper lobe (RUL); 88.9%, right middle lobe (RML); 90.9%, right lower lobe (RLL); 98%, left lower lobe (LUL); 88.9%, left lower lobe (LLL); 98%), lower lobe involvement was seen predominantly during the fourth wave, and middle lobe involvement was the least common (RLL; 53.6%, LLL; 46.4% vs. RUL; 10.1%, RML; 7.2%, LUL; 13%), and it was statistically significant (*p* < 0.001).

**Figure 2:**
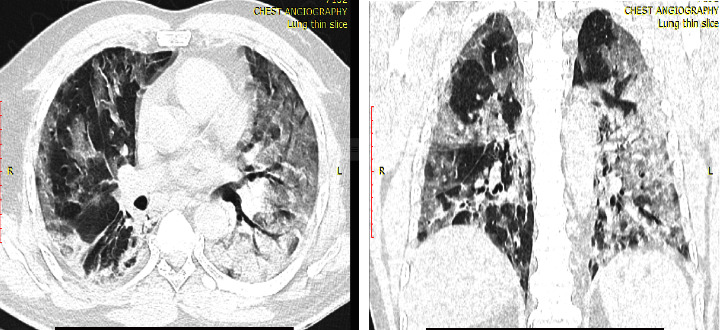
A 45 years old male patient from first wave with bilateral diffuse confluent consolidation and ground glass opacity.

**Figure 3:**
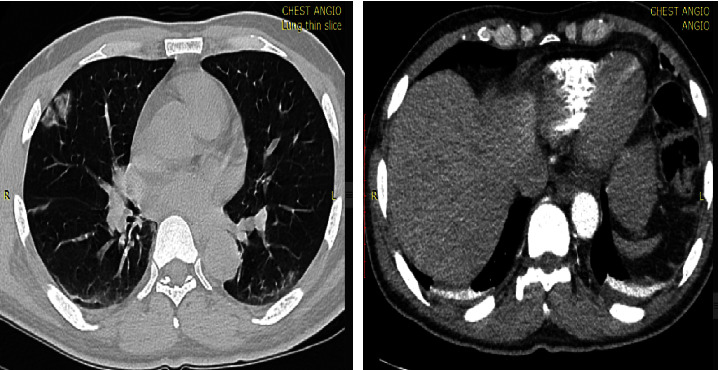
A 60 years old male patient from the fourth wave with peripheral patch ground glass opacity and minimal bilateral pleural effusion.

Ground glass opacity (83.8% vs. 78.3%) followed by lung consolidation (87.9% vs. 56.5%) were the most common chest CT features among patients with positive CT findings in both the first and fourth waves of COVID-19 (Figures 2 and 3). Lung consolidation was more commonly seen among first-wave patients than the fourth wave (*p* < 0.001). Moreover, 10 (10.1%) of the patients during the first wave with chest CT positivity had traction bronchiectasis and halo sign while there were no patients with similar features during the fourth wave. However, there was comparable proportion of patients that presented with broncho-vascular thickening (34.3% vs. 37.7%; *p* = 0.657).

With regard to ground glass opacity, comparably higher percentage of patients presented with GGO in the first wave than fourth wave but it was not statistically significant (*p* = 0.359). In addition, a higher proportion of patients presented with peripheral GGO in the first wave (56.63% vs. 42.59%) while there were higher proportion of central and peripheral GGO in the fourth wave (39.76% vs. 55.56%). Meanwhile, central GGO alone was the least common feature in both waves of COVID-19 among patients that had GGO on chest CT (3.61% and 1.85%). Among patients during the first wave that presented with consolidation on chest CT, a significant majority had peripheral consolidation (74.71%; *p* < 0.001) and hazy margin of consolidation (93.1%; *p* < 0.001). Meanwhile, diffuse consolidation (53.84%) and sharp margin of consolidation (58.97%) were predominantly seen in fourth wave of COVID-19 patients. Central consolidation was the least common feature in both waves (Table 4).

### 3.5. Atypical Chest CT Features among COVID-19 Patients with Positive Findings

In this study, mediastinal lymphadenopathy was more commonly seen among first-wave patients (12.12% vs. 1%) and it was statistically significant (*p* = 0.024). Meanwhile, pleural effusion (*p* < 0.001) and nodular opacity (*p* < 0.001) were predominantly observed among patients from the fourth wave. In addition, there were no patients that presented with tree in bud opacity among first-wave patients while 5 (7.24%) of the study participants from the fourth wave had the feature. There were no patients that had either pneumothorax or cavitation in both waves of COVID-19 (Table 5).

### 3.6. Chest CT Severity Score of COVID-19 Patients during the First and Fourth Waves

The study has revealed that the mean CT severity score for patients during the first wave of COVID-19 was higher than those participants from the fourth wave (13.18 vs. 8.31) and the mean difference was statistically significant (*p* < 0.001) (Table 6).

Regarding CT severity score, there were more than two times higher proportion of patients with severe CT score category among subjects from the first wave than the fourth wave (28% vs. 13%).

Based on age category, the lowest proportion of patients with severe chest CT score was seen among young age groups who are less than the age of 35 years. The study had also revealed females had a higher proportion of patients with severe chest CT score compared to males in both waves. However, there was no statistically significant association between CT severity categories with age and sex. With regards to the duration of symptoms, those patients presenting after two weeks of symptoms had the highest proportion of patients with severe CT score in both waves while those presenting with in the first week had the smallest proportion. Furthermore, the association between duration of symptoms with chest CT severity category was statistically significant (*p* value =0.037) for patients from the first wave of COVID-19.

In this study, comparatively higher proportions of patients with severe CT score category were seen among patients who had comorbidities compared to their counterparts. Meanwhile, there were a comparable proportion of patients among participants who have hypertension and diabetes mellitus in comparison to those who don't in both waves of COVID-19. However, there was no statistically significant association during both waves between CT severity category with age, sex, comorbidity, hypertension, and diabetes mellitus, respectively, in this study (Table 7).

### 3.7. Factors Associated with Chest CT Severity Category among COVID-19 Patients

On bivariable binary logistic regression, only duration of symptom was found to be a statistically significant predictor of CT severity (*p* value =0.038). Those variables with *p* value of <0.25 on bivariable association were included in the multivariable model to adjust for possible confounding effects.

After adjusting for age category and sex, duration of symptom was found to be a statistically significant predictor of chest CT severity during the first wave and those patients that presented after 14 days of symptom onset had 4.12 times higher odd of having severe chest CT score compared to those who presented in the first 7 days of symptom onset (AOR = 4.12; 95% CI: 1.38, 12.27) (Table 8). Binary logistic regression to determine predictor of severity among fourth-wave patients was not done since there was no observed association between severity category and other variables on bivariate association (Table 7).

## 4. Discussion

In this study, CT positivity was higher among patients from the first wave than fourth wave. This could be related to the hypothetical COVID-19 variant during those waves. Our study showed that CT angiography was done during the first wave of COVID more than the fourth wave. This could be due to the more severe feature of the first wave mimicking pulmonary thromboembolism.

The study found that GGO and consolidations were the most common chest CT features among COVID-19 patients during both waves. Furthermore, bilateral lung involvement with lower lobe involvement was predominantly seen in both waves which were in comparative agreement with previous knowledge [9, 19, 20]. Traction bronchiectasis and mediastinal lymphadenopathy, delayed CT features of COVID-19, were seen predominantly among the first-wave patients [9]. This could correlate with the higher proportion of delayed presentation observed during the first wave compared to the fourth wave which allows enough time for the development of fibrosis and bronchiectasis. However, comparatively higher patients with atypical and mixed features were seen during the fourth wave. Previous findings suggested that pleural effusion is an uncommon CT feature among COVID-19 patients [16, 21], but our study found that around a third of patients during the fourth wave had pleural effusion.

It was found that there were statistically significant higher proportions of patients with severe chest CT features in the first wave of COVID-19 compared to the fourth wave. It was also shown that there was a statistically significant mean difference between the two waves with the first wave having a higher mean CT severity score. This could be due to differences in the hypothetical strain of COVID-19 and its virulence. A study done in South Korea had revealed patients with Delta variant had more severe global CT scores compared to those who contracted the omicron variant [22, 23]. The first wave of COVID-19 in Ethiopia was suspected to be related to the alpha variant while the fourth wave was attributed due to omicron [4]. Similar to previous studies, this study had revealed that duration of symptoms was a statistically significant predictor of severity during the first wave of COVID-19 after adjusting for other variables [11, 24]. In comparative agreement with previous studies done, we did not find any association between chest CT severity score and comorbidity status in our study [25, 26].

Chest CT imaging played a crucial role in the diagnosis and management of COVID-19 in both waves of COVID-19 infections by determining severity score and enabling to decide on the mode of management. It was also useful in diagnosing other mimickers like pulmonary thromboembolism. Despite its value, the routine use of chest CT and CT-angiographic studies during COVID-19, especially in resource limited settings, added additional burden to the limited the imaging resources and affected imaging of other diseases, particularly in oncologic patients who do have regular imaging workups with CT.

### 4.1. Strength and Limitation

This study is the first study that tried to explore differences in CT imaging between different waves of COVID-19 infection. However, this study was not without limitations. Firstly, this study utilized secondary data and lacks important variables including smoking history and vaccination status, re-infection status. Secondly, the study included subjects from purposively selected two diagnostic centers which would limit the generalizability of the result. Finally, features of pulmonary thromboembolism and COVID-19 on chest CT without contrast can be similar. This could have led to misclassification and overestimation of the magnitude of chest CT positivity.

## 5. Conclusion and Recommendation

The study has revealed that chest CT positivity was comparatively higher for first-wave patients. Common chest features included bilateral involvement, lower lobe involvement, and consolidation. The mean chest CT severity was comparatively higher for first wave than fourth wave, and the duration of symptoms was a statistically significant predictor of CT severity for first wave.

We recommend further research with variables such as smoking history and immunization history included as well as a study with a larger sample size involving multiple diagnostic center.

## Figures and Tables

**Table 1:** Sociodemographic and comorbidity profile of patients during the 1^st^ and 4^th^ wave COVID-19 in Addis Ababa city.

Variables	1^st^ wave	4^th^ wave
	Frequency (%), *n* = 100	Frequency (%), *n* = 100
Age		
<35 years	16 (16%)	15 (15%)
36–50 years	41 (41%)	23 (23%)
>50 years	43 (43%)	62 (62%)
Sex		
Male	53 (53%)	69 (69%)
Female	47 (47%)	31 (31%)
Comorbidity		
Yes	44 (44%)	63 (63%)
No	56 (56%)	37 (37%)
Number of comorbidities		
Single comorbidity	36	27
Multiple comorbidities	8	36
Hypertension	23 (23%)	30 (30%)
Underlying lung disease	2 (2%)	11 (11%)
DM	22 (22%)	29 (29%)
HIV/AIDS	4 (4%)	3 (3%)
Renal disease	1 (1%)	7 (7%)
Cardiac illnesses	0 (0%)	16 (16%)
Malignancy	0 (0%)	9 (9%)

**Table 2:** Symptom profiles of study participant COVID-19 patients.

Variables	1^st^ wave	4^th^ wave	*p* value
	Frequency (%), *n* = 100	Frequency (%), *n* = 100	
Duration of symptoms			0.075
1–7 days	59 (59%)	74 (74%)	
8–14 days	14 (14%)	10 (10%)	
>14 days	27 (27%)	16 (16%)	
Cough	76 (76%)	82 (82%)	0.298
Headache	12 (12%)	4 (4%)	0.037
Shortness of breath	63 (63%)	49 (49%)	0.064
Arthralgia/myalgia	3 (3%)	33 (33%)	<0.001
Fever	18 (18%)	18 (18%)	1
Loss of taste/smell	4 (4%)	2 (2%)	0.683
Chest pain	33 (33%)	17 (17%)	0.014
Sore throat	2 (2%)	49 (49%)	<0.001
Fatigue	22 (22%)	10 (10%)	0.034

**Table 3:** Comparison of chest computed tomography (CT) finding of COVID-19 patients during the 1^st^ and 4^th^ waves COVID in Addis Ababa city.

Variables	1^st^ wave	4^th^ wave	*p* value
	Frequency (%)	Frequency (%)	
Chest CT protocols			<0.001
Chest angiography	61 (61%)	50 (40%)	
CT without contrast	26 (26%)	30 (30%)	
CT with contrast	13 (13%)	20 (20%)	
CT findings			
Positive	99 (99%)	69 (69%)	<0.001
Unremarkable	1 (1%)	31 (31%)	

**Table 4:** Chest CT imaging patterns among COVID-19 patients with positive imaging findings.

Variables	1^st^ wave	4^th^ wave	*p* value
	Frequency (%), *n* = 99	Frequency (%), *n* = 69	
Number of features			0.095
Single features	12 (12.1%)	15 (21.7%)	
Multiple features	87 (87.9%)	54 (78.3%)	
Symmetry of involvement			0.021
Unilateral	2 (2%)	7 (10.1%)	
Bilateral	97 (98%)	62 (89.9%)	
Zonal distribution			0.004
Central	2 (2%)	7 (10.1%)	
Peripheral	58 (58.6%)	25 (36.2%)	
Diffuse	39 (39.4%)	37 (53.6%)	
Lobar involvement			
Right upper lobe	88 (88.9%)	7 (10.1%)	<0.001
Right middle lobe	90 (90.9%)	5 (7.2%)	<0.001
Right lower lobe	97 (98%)	37 (53.6%)	<0.001
Left upper lobe	88 (88.9%)	9 (13%)	<0.001
Left lower lobe	97 (98%)	32 (46.4%)	<0.001
Ground glass appearance(GGO)	83 (83.8%)	54 (78.3%)	0.359
Central	3 (3.61%)	1 (1.85%)	
Peripheral	47 (56.63%)	23 (42.59%)	
Central and peripheral	33 (39.76%)	32 (55.56%)	
Broncho-vascular thickening	34 (34.3%)	26 (37.7%)	0.657
Crazy paving	6 (6.1%)	16 (23.2%)	0.001
Traction bronchiectasis	10 (10.1%)	0 (0%)	
Halo sign	10 (10.1%)	0 (0%)	
Consolidation	87 (87.9%)	36 (52.2%)	<0.001
Central	1 (1.15%)	4 (10.26%)	
Peripheral	65 (74.71%)	14 (35.9%)	
Diffuse	21 (24.14%)	21 (53.84%)	
Margin of consolidation			<0.001
Sharp	4 (4.1%)	23 (33.8%)	
Hazy	81 (81.8%)	12 (17.7%)	
Not applicable	14 (14.1%)	33 (48.5%)	
Multiple round consolidations	7 (8.04%)	16 (23.2%)	0.02

**Table 5:** Atypical chest computed tomography (CT) features among COVID-19 patients with positive CT findings.

Variables	1^st^ wave	4^th^ wave	*p* value
	Frequency (%), *n* = 99	Frequency (%), *n* = 69	
Mediastinal lymphadenopathy	12 (12.12%)	1 (1.45%)	0.024
Nodular opacity	3 (3.03%)	17 (24.6%)	<0.001
Pleural effusion	11 (11.11%)	33 (47.8%)	<0.001
Tree in bud opacity	0 (0%)	5 (7.24%)	

**Table 6:** Independent sample *t*-test to compare mean CT severity scores between 1^st^ and 4^th^ waves of COVID-19.

Variable	1^st^ wave	4^th^ wave	*t*-test for equality of means			
			*T*	df	*p* value	95% CI for mean difference
Mean CT score (SD)	13.18 (6.29)	8.31 (7.53)	5.06	198	<0.001^*∗*^	(3.015, 6.865)

*p* ^*∗*^ < 0.05.

**Table 7:** Comparison of Chest CT severity features among the sociodemographic and comorbidity profiles.

Variables	1^st^ wave, *n* = 100			4^th^ wave, *n* = 100		
	Severe, *n* = 28 (28%)	Nonsevere, *n* = 72 (72%)	*p* value	Severe, *n* = 13 (13%)	Nonsevere, *n* = 87 (87%)	*p* value
Age						
<35	1 (6.2%)	15 (93.8%)	0.107	2 (13.3%)	13 (86.7%)	0.758
36–50	13 (31.7%)	28 (68.3%)		4 (17.4%)	19 (82.6%)	
>50	14 (32.6%)	29 (67.4%)		7 (11.3%)	55 (88.7%)	
Sex						
Male	12 (22.6%)	41 (77.4%)	0.205	8 (11.6%)	61 (88.4%)	0.533
Female	16 (34%)	31 (66%)		5 (16.1%)	26 (83.9%)	
Duration of symptoms						
1–7 days	11 (18.6%)	48 (81.4%)	**0.037** ^*∗*^	7 (9.5%)	67 (90.5%)	0.193
8–14 days	5 (35.7%)	9 (64.3%)		2 (20%)	8 (80%)	
>14 days	12 (44.4%)	15 (55.6%)		4 (25%)	12 (75%)	
Comorbidity						
Yes	13 (29.5%)	31 (70.5%)	0.76	11 (17.5%)	52 (82.5%)	0.317
No	15 (26.8%)	41 (73.2%)		2 (5.4%)	35 (94.6%)	
Diabetes mellitus						
Yes	6 (27.3%)	16 (72.7%)	0.931	4 (13.8%)	25 (86.2%)	1.00
No	22 (28.57%)	55 (71.43%)		9 (12.6%)	62 (87.4%)	
Hypertension						
Yes	7 (30.4%)	16 (69.6%)	0.767	4 (13.3%)	26 (86.7%)	1.00
No	21 (27.3%)	56 (72.7%)		9 (12.9%)	61 (87.1%)	

*p* ^*∗*^ < 0.05; the bold value represent statistical significance.

**Table 8:** Predictors of chest CT severity category among COVID-19 patients during the first wave of COVID-19.

Variables	COR	95% CI	AOR	95% CI	*p* value
Age					0.177
<35 years	1		1		
36–50 years	6.964	0.829, 58.51	7.879	0.887, 69.94	0.064
>50	7.241	0.867, 60.47	5.745	0.658, 50.14	0.114
Sex					
Male	0.567	0.235, 1.37	0.441	0.159, 1.224	0.116
Female	1		1		
Duration of symptoms					**0.038** ^*∗*^
1–7 days	1		1		
8–14 days	2.424	0.678, 8.671	2.002	0.524, 7.64	0.31
>14 days	3.491	1.281, 9.515	4.12	1.38, 12.27	**0.011** ^*∗*^

^*∗*^ *p* < 0.05; bold values indicate statistically significant associations.

## Data Availability

The data used to support the findings of this study are available from the corresponding author upon request.
